# National experience with adenosine deaminase deficiency related SCID in Polish children

**DOI:** 10.3389/fimmu.2022.1058623

**Published:** 2023-01-06

**Authors:** Nel Dąbrowska-Leonik, Barbara Piątosa, Ewa Słomińska, Nadezda Bohynikova, Katarzyna Bernat-Sitarz, Ewa Bernatowska, Beata Wolska-Kuśnierz, Krzysztof Kałwak, Sylwia Kołtan, Anna Dąbrowska, Jolanta Goździk, Marek Ussowicz, Małgorzata Pac

**Affiliations:** ^1^ Department of Immunology, Children’s Memorial Health Institute, Warsaw, Poland; ^2^ Histocompatibility Laboratory, Children’s Memorial Health Institute (IPCZD), Warsaw, Masovian, Poland; ^3^ Biochemistry Department, Medical University of Gdansk, Gdansk, Poland; ^4^ Department of Paediatric Bone Marrow Transplantation, Oncology and Hematology, Wroclaw Medical University, Wroclaw, Poland; ^5^ Department of Pediatrics, Hematology and Oncology, Faculty of Medicine, Nicolaus Copernicus University in Toruń, Bydgoszcz, Poland; ^6^ Department of Clinical Immunology and Transplantology, Faculty of Medicine, Jagiellonian University Medical College, Kraków, Poland

**Keywords:** adenosine deaminase (ADA) deficiency, SCID - severe combined immunodeficiency, ERT (enzyme replacement therapy), HSCT = hematopoietic stem cell transplant, lymphopenia

## Abstract

**Introduction:**

Deficiency of adenosine deaminase (ADA) manifests as severe combined immunodeficiency (SCID), caused by accumulation of toxic purine degradation by-products. Untreated patients develop immune and non-immune symptoms with fatal clinical course. According to ESID and EBMT recommendations enzyme replacement therapy (ERT) should be implemented as soon as possible to stabilize the patient’s general condition, normalize transaminases, treat pulmonary proteinosis, bone dysplasia, and protect from neurological damage. Hematopoietic stem cell transplantation (HSCT) from a matched related donor (MRD) is a treatment of choice. In absence of such donor, gene therapy (GT) should be considered. HSCT from a matched unrelated donor (MUD) and haploidentical hematopoietic stem cell transplantation (hHSCT) are associated with worse prognosis.

**Material and methods:**

We retrospectively evaluated the clinical course and results of biochemical, immunological and genetic tests of 7 patients diagnosed in Poland with ADA deficiency since 2010 to 2022.

**Results:**

All patients demonstrated lymphopenia affecting of T, B and NK cells. Diagnosis was made on the basis of ADA activity in red blood cells and/or genetic testing. Patients manifested with various non-immunological symptoms including: lung proteinosis, skeletal dysplasia, liver dysfunction, atypical hemolytic-uremic syndrome, and psychomotor development disorders. Five patients underwent successful HSCT: 3 patients from matched unrelated donor, 2 from matched sibling donor, and 1 haploidentical from a parental donor. In 4 patients HSCT was preceded by enzyme therapy (lasting from 2 to 5 months). One patient with multiple organ failure died shortly after admission, before the diagnosis was confirmed. None of the patients had undergone gene therapy.

**Conclusions:**

It is important to diagnose ADA SCID as early as possible, before irreversible multi-organ failure occurs. In Poland HSCT are performed according to international immunological societies recommendations, while ERT and GT are less accessible. Implementation of Newborn Screening (NBS) for SCID in Poland could enable recognition of SCID, including ADA-SCID.

## Introduction

Adenosine deaminase (ADA) deficiency is a rare inherited disorder of purine metabolism leading to life threatening severe combined immunodeficiency (SCID), otherwise known as ADA-SCID (1). The disease is inherited in an autosomal recessive manner, with incidence 1:200 000 - 1: 1 million births, and accounts for 10-20% of all SCID cases. The accumulation of deoxyadenosine, a metabolic substrate of ADA enzyme, causes abnormalities in immune cells development and functions and has systemic impact, mostly on skeletal development (costochondral abnormalities, skeletal dysplasia), cerebral (cognitive and behavioral defects), as well as hepatic and pulmonary functions (1, 2).

Most affected infants present with severe opportunistic infections, failure to thrive and developmental delay during their first 6 months of life. Undiagnosed or left untreated children die in their first year of life.

The hematopoietic stem cell transplantation (HSCT) from matched related donor (MRD) represents a successful treatment option with high survival rates and excellent immune reconstitution, and is an accepted treatment of choice. Two other therapeutic options are available: enzyme replacement therapy (ERT) and gene therapy (GT) (1).

This study reviews the diagnostic and therapeutic approach to ADA-SCID.

## Materials and methods

This study included 7 Caucasian children 2 girls and 5 boys, now aged from 6 months to 13 years, diagnosed with ADA-SCID in Poland since 2010 to early 2022. Five patients were diagnosed in the Department of Immunology, Children’s Memorial Health Institute (CMHI), Warsaw, two others – in the Department and Clinic of Pediatric Oncology, Haematology and Bone Marrow Transplantation, Wroclaw Medical University, Wroclaw, Poland and in the Department of Paediatrics, Haematology and Oncology, Collegium Medicum in Bydgoszcz, Nicolaus Copernicus University in Torun, respectively. We retrospectively evaluated the clinical course and results of biochemical, immunological, and genetic tests leading to the diagnosis of ADA-SCID. Basic hematological, biochemistry, and immunological results, including immunoglobulin level, lymphocyte proliferation test, and flow cytometry results were recorded.

Adenosine deaminase (ADA) activity was measured in patients’ erythrocyte lysate and expressed in nmol/h/mg Hb (3).

Molecular diagnostics involved whole gene direct Sanger sequencing in 3 cases, and next-generation sequencing (NGS) for primary immunodeficiency gene panel with confirmatory Sanger sequencing to targeted region in 4 patients.

Evaluation of lymphocyte subsets was performed using commercially available BD Multitest six color cocktails of antibodies and Trucount tubes (Becton Dickinson, cat. no. 644611). The assessed lymphocyte subset panel included T cells (CD3+/CD45+), cytotoxic T cells (CD3+CD8+/CD45+), helper T cells (CD3+CD4+/CD45+), NK cells (CD16+CD56+CD3-/CD45+), and B cells (CD19+/CD45+). Their distribution and absolute cell counts were determined using the lyse-no-wash approach according to manufacturer’s instructions (4). The absolute number of individual subsets was calculated based on the proportion of the respective cell subpopulation and absolute lymphocyte count.

ERT was carried out in 4 patients in the Department of Immunology, Children’s Memorial Health Institute, Warsaw. One patient received polyethylene glycol-conjugated bovine ADA, intramuscularly at a weekly dose of 20 U/kg and 3 patients were treated with elapegademase, at a dose 0.4 mg/kg a week in 1-2 doses. Six patients underwent HSCT in three Polish centers.

The study was performed in accordance with the Declaration of Helsinki on Biomedical Research involving Human subjects. The study was approved by the Bioethics Committee at the Children’s Memorial Health Institute (Decision No. 21/KBE/2022).

## Results

**Figure 1:**
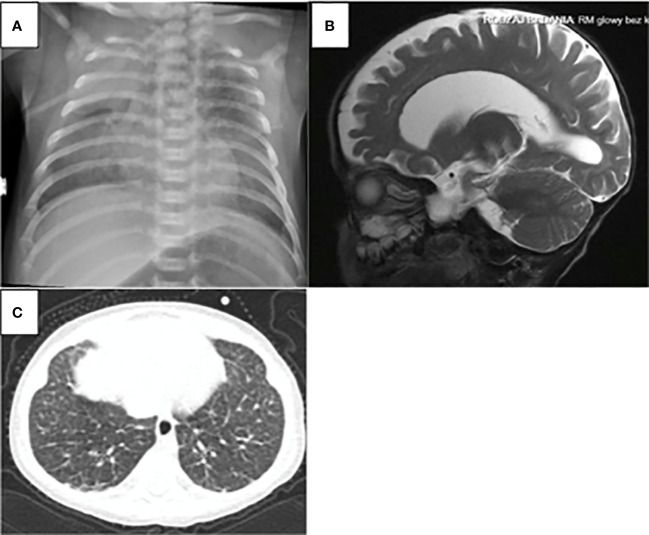
ADA-SCID sypmtoms: **(A)** Chest X-ray: scapular spurring with flaring and cupping of rid ends in the patient 6. **(B)** atrophic changes in the CNS were found in the brain magnetic in the patient 3 **(C)** computed tomography of the chest as ground-glass opacities with interlobular and intralobular interstitial thickening in patient 5.

The diagnosis of ADA SCID was made in all 7 patients at the median age of 4.5 months (ranged 7 weeks to 27 months). None of the patients had positive family history or was detected in the Polish-German transborder area “RareScreen” newborn screening project (5). According to available data between 2010 and February 2022 SCID was diagnosed in 64 patients in Poland. The prevalence of ADA-SCID among all SCID was 10.9%. The first symptoms appeared during the first month of life in 4 patients (57%). Pneumonia was observed in all patients, in 4 of them during first months of life (2 weeks to 3 months of age), while in 3 others after the first year of life. Lymphopenia was diagnosed in 2 patients (28.5%) and failure to thrive in 2 other children (28.5%). Only 2 patients (P6, P7) demonstrated skeletal abnormalities on the chest X-ray as scapular spurring with flaring and cupping of rib ends (**Figure 1**).

Elevated transaminases were observed in total in 5 patients (71%), in 3 of them at diagnosis (43%). In patient P5, transaminase activity was slightly elevated already at the time of diagnosis, but significant increase in the value of transaminases (Alanine Aminotransferase - ALT 331 U/l, Aspartate Aminotransferase - AST 902 U/l) was observed while waiting for the ERT.

Delay in diagnosis ranged from 1.5 to 15 months (**Table 1**). Chronologically, first 3 patients were diagnosed after first year of life and the next 4 -in the first 6 months of life (**Table 1**)]. In patient P3, SCID was not suspected until at the age of 15 months due to severe nephrological symptoms i.e. atypical hemolytic uremic syndrome (aHUS) and arterial hypertension. At the time of the diagnosis the patient demonstrated multiple opportunistic infections (cytomegalovirus infection, *Pneumocystis jiroveci* and *Absidia* pneumonia) and advanced atrophic changes in the Central Nervous System (CNS) found in the brain magnetic resonance imaging. He died prior to establishing ADA-SCID diagnosis and was not treated with ERT or HSCT (6).

**Table 1:** Age of onset of symptoms and diagnosis.

Pt	Sex	Lymphopenia	Neutropenia	Hypertransaminasemia	Pneumonia	Failure to thrive	Hepatomegaly	Bone dysplasia	Infections	Age at SCID diagnosis	Age at ADA diagnosis
P1	F	15 mo	13 mo	15 mo	6 mo	15 mo	No	No	No	13 mo	21 mo
P2	M	15 mo	15mo	15 mo	2x	x	16 mo	No	2.5y CMV, Asp	27 mo	27 mo
P3	M	14 mo	15 mo	15 mo	15 mo	since birth	15 mo	No	3 mo, VZV 9 mo HSV 15 mo CMV, PJP	15 mo	16 mo
P4	M	2.5 mo	2.5 mo	2,5 mo	3 mo	since birth	6 mo	No	No	3 mo	6 mo
P5	F	7 w	7 w	7 w	7 w	2 m	No	No	Local BCG 5 mo Can	2 mo	3 mo
P6	M	2 w	2 w	3 w	2 w	no	No	2 mo: X-chest	Local BCG 2 mo RSV, Can	7 w	8 w
P7	M	5 w	5 w	7 w	5w	no	No	2 mo: X-chest	5 w PJP, ADV Local BCG 3 mo, Can	6 w	7 w

ADA, adenosine deaminase; ADV, adenovirus; Asp, Aspergillus spp; BCG, Bacille Calmette-Guerin; Can, Candida spp; CMV, cytomegalovirus; F, female; HSV, herpes simplex virus; M, male; Mo, months; PJP, Pneumocystis jiroveci pneumonia; RSV, respiratory syncytial virus; Pt, patient; SCID, severe combined immunodeficiency; w, weeks; VZV, Varicella Zoster Virus.

**Figure 2:**
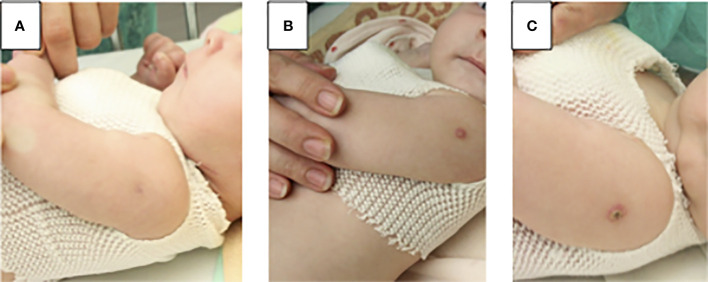
Local BCG disease in patient 5: **(A)** before enzyme replacement therapy (ERT) normal BCG vaccination scar **(B)** 4 weeks of ERT, at the site of the BCG injection small spot appeared and turned into a blister, treatment with two tuberculostatic drugs: isoniazid and rifampicin has started **(C)** & weeks of ERT, a crusty scrab formed, which healed into a small scar.

We confirmed viral infections in 4 patients (57%): 2 had cytomegaloviral infection, 1 respiratory syncytial virus (RSV), and 1 adenoviral (ADV) infection, 1 varicella zoster virus (VZV), and herpes simplex (HSV) (**Table 1**). Fungal infections were suspected in 4 more patients apart from patient 3. In 3 patients *Candida* infection was suspected due to the presence of fungal metabolites in serum (mannan or beta-D-glukan), in 1 patient *Aspergillius* (galactomannan in the serum), and in 2 patients *Pneumocystis jiroveci* infection (positive specific IgG, chest x-ray image) (**Table 1**). Local Bacillus-Calmette-Guerin (BCG) complication was observed in 3 patients. In 2 of them local inflammation at the site of vaccination without lymphadenopathy developed between 3^rd^ day and 4th week after initiation of ERT preliminary. In P5 the first signs of BCG complication were observed after 4 weeks of ERT and disappeared after starting treatment with two tuberculostatic drugs: isoniazid and rifampicin (**Figure 2**).

Laboratory tests showed lymphopenia in all patients, affecting subpopulations of T, B and NK cells (**Table 2**).

**Table 2:** Laboratory and clinical data at diagnosis.

Pt	WBC (K/μl)	ALC (K/μl)	AST (U/l)	ALT (U/l)	IgG (g/l)	IgA (g/l)	IgM (g/l)	CD3 (cells/ml)	CD4 (cells/μl)	CD8 (cells/μl)	CD19 (cells/μl)	CD16+ 56+CD3- (cells/μl)	RTE (%)	ADA activity (nmol/mg Hb/h)	Gene defect
P1	2,64 (L)	0.21 (L)	85 (H)	59 (H)	3.39 (N)	0.08 (N)	0.315 (N)	65 (L)	42 (L)	21 (L)	14 (L)	70 (L)	Nd	N	c.80T>A(;)646G>A p.Ile27Asn(;)Gly216Arg
P2	2 (L)	0.58 (L)	180 (H)	141 (H)	3.84 (L)	0.41 (N)	0.39 (N)	714 (L)	107 (L)	618 (L)	24 (L)	25 (L)	5.4 (L)	0.63 (L)	Not detected
P3	1.9 (L)	0.34 (L)	56 (H)	21 (N)	4.75 (N)	<0.06 (L)	<0.04 (L)	50 (L)	1 (L)	49 (L)	1 (L)	11 (L)	0 (L)	Nd	homozygous mutation c.302G>A p.Arg101Gln
P4	1.56 (L)	0.17 (L)	225 (H)	38 (N)	3.9 (N)	0.4 (N)	1.11 (N)	39 (L)	33 (L)	2 (L)	42 (L)	17 (L)		Nd	homozygous mutation c.302G>A p.Arg101Gln
P5	1.7 (L)	0.25 (L)	133 (H)	51 (N)	6.92 (H)	0.36 (N)	1.32 (H)	158 (L)	71 (L)	73 (L)	3 (L)	64 (L)	9.8 (L)	0.109 (L)	c.302G>A(;)956_960delAAGAG p.Arg101Gln(;)Glu319Glyfs
P6	3.12 (L)	0.14 (L)	67 (N)	31 (N)	8.54 (4 days IVIG)	0.07 (N)	<0.04 (L)	35 (L)	31 (L)	4 (L)	2 (L)	5 (L)	0 (L)	4.9 (5 days after transfusion) (L)	c.363-3C>G(;)778G>A p.(?)(;)Glu260Lys
P7	1.22 (L)	0.04 (L)	67 (N)	29 (N)	2.1 (L)	<0.07 (N)	0.08 (L)	27 (L)	20 (L)	7 (L)	3 (L)	22 (L)	0 (L)	0 (L)	c.956_960del(;)del ex 2: chr20: 44636227-44636288 p.Glu319Glyfs(;)

ADA, adenosine deaminase; ALC , absolute lymphocyte count; ALT, alanine transaminase; AST, aspartate aminotransferase; CD, cluster of differentiation; IgA, immunoglobulin A; IgG, immunoglobulin G; IgM, immunoglobulin M; L, below norm; N, normal; Nd, not done; H, above normal; RTE, Recent Thymic Emigrants: CD31+CD45RA+/CD3+CD4+; WBC, white blood cells.

The diagnosis was made by measurement of ADA activity in 4 patients and in 6 patients confirmed by genetic testing. ADA activity in red blood cells (RBC) was very low in 3 patients (<1%) and low (4.9%) in one patient, who was tested 5 days after RBC transfusion (**Table 2**).

Genetic tests revealed the presence of 7 mutations in 6 patients. The most common variant (c.302G> A) was detected in 3 patients: in P3 and P4 as homozygous mutation while in P5 as heterozygous. The next common one (c.956_960delAAGAG) was found in 2 patients (P5, P7) in heterozygous genotype. In the rest of patients other variants were found (**Table 2**). In patient P2 no mutation was found, but testing was done only by Sanger.

**Figure 3:**
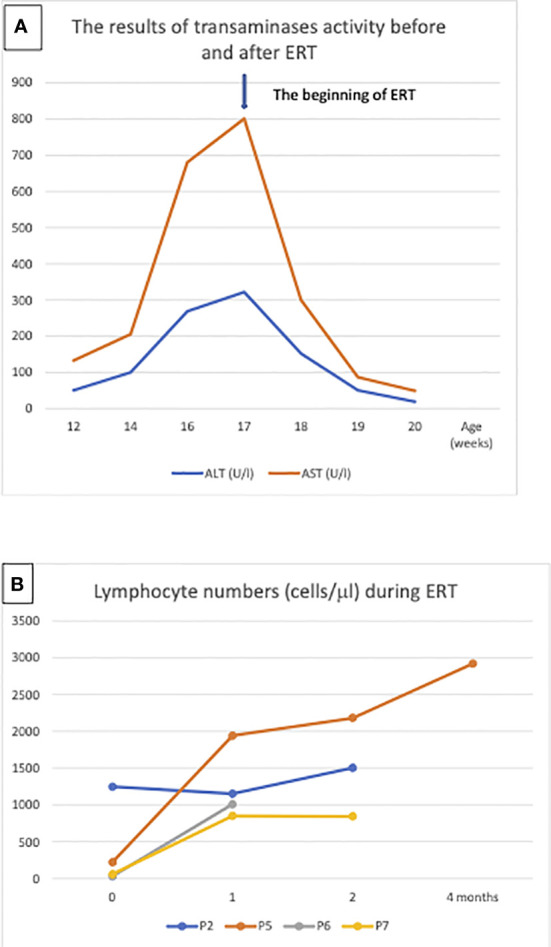
Enzyme replacement therapy (ERT): **(A)** Aminotransferases normalization in patient 5 after ERT, the arrow indicates the beginning of treatment, **(B)** Lymphocyte numbers in patient P2, P5, P6 and P7 after 1, 2 and 4 months ERT.

ERT was administered to 4 patients for the period of 6 to 23 weeks with normalization of the lymphocyte count and transaminases activity (**Figure 3**).

HSCT was performed during the period of 2010-2022 in 6 patients: 2 MSD, 3 MUD, 1 haploidentical parental donor. All patients are alive at the time of preparing of this manuscript. The conditioning regimens in 5 cases were used in accordance with successive ESID recommendations. In two cases the regimen included treosulfan, fludarabine, and thiotepa.

None of the patients was referred for gene therapy (GT), which is available only in two centers across Europe. According to Polish regulations, the patient should obtain an agreement of the Ministry of Health for that treatment. Among five patients of CMHI one had a MSD, one died before any treatment and, only one agreed to undergo GT, however the parents eventually refused, due to complicated family situation. Two other patients decided to go for HSCT.

## Discussion

The diagnosis of ADA SCID was established after typical symptoms appeared in the first months of life. The reported patients most frequently suffered from dyspnea with radiographical pneumonia, unresponsive to conventional treatment and leukopenia. Leukopenia and dependence on oxygen therapy stimulated the diagnostic work up for a primary immunodeficiency.

Pulmonary manifestation was demonstrated on imaging studies as ground-glass opacities with interlobular and intralobular interstitial thickening (**Figure 1**). These findings might be associated with infections, but they also may result from pulmonary alveolar proteinosis (PAP) frequently observed in the patients with ADA deficiency. Lung biopsy or bronchoalveolar lavage that could confirm pulmonary proteinosis were not performed, but improvement was observed with ERT treatment, with subsequent disappearance of dyspnea or weaning of oxygen therapy in two patients (P5, P6). According to the literature, reverse of PAP is observed after enzyme replacement therapy or after transplantation (7).

Although skeletal manifestations of ADA SCID, like scapular squaring and spurring and cupping of the costochondral junctions were not the first manifestation in Polish patients, they were observed in 2 patients after establishment of the diagnosis (8). Hypertransaminasemia was observed in the of patients. Patient P5 demonstrated significant increase in the value of transaminases during the period preceding initiation of ERT. Considering that hepatic failure can be rapid in patients with ADA-SCID, urgent treatment was required (9). RBC transfusions, intended to provide the child temporarily with external source of ADA enzyme, and reduction of antibiotic prophylaxis were not effective in this patient. Significant improvement was achieved after initiation of ERT (**Figure 3**).

One of the reported patients (P2) showed mild phenotype with lung and liver function abnormalities but no life-threatening infections. Symptoms in this patient related to recurrent pneumonia showed milder course than in patients diagnosed with SCID, but hepatosplenomegaly, and hypertransaminasemia were present, and the child was consulted by pulmonologist and gastroenterologist before final diagnosis was established. Diagnosis of leaky ADA-SCID in patient P2 was based on criteria published by Shearer et al. (10), defined as lymphopenia CD3 T cells below 1000 cells/µl for the age up to 2 years, absence of maternal engraftment, and less than 30% of lower limit of normal T cell function (as measured by response to phytohemagglutinin (PHA). Delay in the diagnosis was 12 months from first symptoms, which emphasizes the need for multi-specialist teams taking care of children with rare diseases.

Another challenge in the reported group, was to initiate the right diagnostic approach in patients with atypical symptoms. In patient P3, recurrent diarrhea from the first month of life, failure to thrive and delayed psychomotor development were not sufficiently indicative of IEI. Even infections with VZV (at 3 months) and HSV (at 9 months) were not associated with immunodeficiency. Atypical hemolytic uremic syndrome (aHUS), respiratory failure, requirement for peritoneal dialysis and plasmapheresis, finally led to initiation of the diagnostic procedure for an inborn error of immunity. At the age of 15 months, when the diagnosis of SCID was finally established, the patient had already signs of multiple organ failure (MOF), advanced atrophic changes in the CNS, severe arterial hypertension, cytomegalovirus infection, and *Pneumocystis jiroveci* and *Absidia* pneumonia. Diagnosis of ADA-SCID was established *post mortem* (6).

Another issue that needs addressing here, is the post-BCG vaccination disease in an IEI patient (11). In Poland, vaccination against tuberculosis is obligatory in the first two days of life in all newborns, unless they have contraindications. For this purpose, locally-produced BCG Moreau strain vaccine is used, a descendant of the Brasilian BCG Moreau substrain with superior clinical safety profile. Serious complications in IEI patients are only observed in SCID, especially associated with low NK cell numbers, and patients with Mendelian susceptibility to mycobacterial diseases (MSMD). NK cells are thought to have a protective role probably due to IFN-γ production (11). However, none of our patients with ADA-SCID demonstrated a disseminated BCG disease, with local manifestations observed in total in 3 patients, one before ERT and two after initiation of ERT and with an increase in the number of lymphocytes, treated with antimycobacterial double therapy (isoniazid, rifampicin) (**Figure 2**; **Table 3**).

**Table 3:** ERT and HSCT characteristics and outcomes.

Pt	Age at ERT onset	ERT duration (weeks)	Age at HSCT (mo)	Donor	Type of conditioning	CD34+/kgx10^6^	Clinical complications	Post-HSCT follow up (month)	Last lymphocyte value (K/ul)	Last engraftment (CD3, CD19, PMN)	BCG treatment
P1	n.a.	n.a.	16	MSD	none	6.55	None	140	2860	1897/261/4480	n.a.
P2	28 mo	12	33	MSD	Bu-Flu	4.28	hypertensive crisis, FUO, CMV	131	2350	1746/413/1450	n.a.
P3	n.a.	n.a.	n.a.	n.a.	n.a.	n.a.	n.a.	n.a.	n.a.	n.a.	n.a.
P4	no	no	7	MUD	myeloablative, toxicity reduced (TREO+FLUD)	4.04	no	72	1900	1362/218/3750	n.a.
P5	4 mo	23	9	MUD	BuxFlu	4.6	mucositis III stage, hypertension	46	4600	chimerism 12-2021 - 88%	INH+RMP
P6	11 w	6	4	Hapl	Treo-flu-tiotepa	18.72	mucositis aGVHD st.2, autoimmune hemolytic anemia, patient retransplanted from the same donor (08.2022)	18		n/a	INH+RMP
P7	10 w	9	4,5	MUD	Treo-flu-tiotepa		aGVHD stage 2, autoimmune hemolytic anemia	3	600	475/0/2370	INH+RMP

aGVHD, acute graft versus host disease; BCG, Bacille Calmette-Guerin; CD, cluster of differentiation; CMV, cytomegalovirus; ERT, enzyme replacement therapy; FUO, fever of unknown origin; HSCT, hematopoietic stem cell transplantation; INH, isoniazid (isonicotinic acid hydrazide); mo, months; MSD, matched sibling donor; MUD, matched unrelated donor; n.a., not applicable; RMP, rifampicin; w, weeks.

Currently, ADA-SCID diagnostics in Poland is quite easily accessible and not expensive. In cases when typical clinical manifestations are accompanied by poor flow cytometry results suggesting ADA-SCID diagnosis, the activity of the ADA enzyme in red blood cells is evaluated, and results confirmed by molecular methods. Before 2010, for various reasons the delay in the diagnostic process was far too long, leading frequently severe complications as in case of first patients from the described group, diagnosed at the age between 16 and 27 months, or fatal outcome without diagnosis. Therefore, the exact number of ADA SCID patients in Poland before 2010 remains unknown. The prevalence of ADA-SCID among all SCID diagnosed between 2010 and 2022 was 10.9%. According to the Statistics Poland reports during this period 50-88 infants’ deaths were noted yearly due to different infections (Archive on the website: https://stat.gov.pl/en/topics/statistical-yearbooks/statistical-yearbooks/demographic-yearbook-of-poland-2021,3,15.html). It can’t be excluded that some undiagnosed SCID patients were among them.

The newborn screening program for SCID could enable identification of such infants. In Polish-German transborder NBS study (TREC and KREC assays) among 101 012 tested newborns (37 025 from Poland and 63 987 in Germany) two cases of atypical SCID/CID (both in Germany), two cases of autosomal recessive agammaglobulinemia (homozygous mutations in IGLL1 gene – one in Germany, one in Poland), one case of Nijmegen breakage syndrome (in Poland) were diagnosed. No ADA-SCID patient was detected, probably because of small group of newborns.

Standard curative option of ADA-SCID treatment, HSCT from MSD, is offered in Poland for children with HLA-matched sibling as a first choice (12). Based on data on HSCT performed between 1981 and 2009 in 16 centers worldwide, an overall survival rate (OS) in ADA-SCID depends on type of HSCT, being 86% for MSD, 81% for MFD, 66% for MUD and 43 for Haplo (13). Recent studies on large groups of patients: 131 ADA-SCID diagnosed in 1982-2017 (14) and 152 patients with SCID, including 43 ADA-SCID treated in 2006-2014 (15), showed higher OS in patients transplanted from MSD/MFD under 3.5 months of age and without active infections. According to the European Society for Immunodeficiencies (ESID), the European Society for Blood and Marrow Transplantation (EBMT), and European Reference Network on Rare Primary Immunodeficiency Autoinflammatory Autoimmune diseases (RITA), gene therapy should be considered in absence of a matched family donor (16). This therapeutic modality does not require donor search and it is associated with lower incidence of complications, including absence of graft versus host disease (GvHD) and procedure-related mortality. Limited availability and lack of reimbursement are the major obstacles for GT in Poland - it is available in only very few centers in Europe, but not in Poland, and the cost is extreme. Besides, longer duration of the procedure and time to achieve immune reconstitution are important clinical drawbacks. Frequency of therapy failure is similar: 10-20% in MSD HSCT and 5-20% in GT, but procedure-related mortality is 5.6 and 0% respectively (12). Some authors recommend considering GT as a first-line treatment, even if MSD is available, as this procedure abrogates any risk of alloreactivity (17). However, since treosulfan-based conditioning was introduced results for MUD, and Haplo HSCT are comparable with MSD in some centers and therefore recommended when there is no access to GT (18). GT is not readily available in Poland due to lack of specialized treatment center, as well as high cost of the procedure and need to run ERT until GT is carried out.

ERT is an effective treatment that has been used for over 3 decades. It should be implemented as soon as possible to stabilize the patient’s general condition, detoxify the organism from accumulating purine metabolites, normalize transaminases, as well as treat pulmonary alveolar proteinosis and bone dysplasia. ERT is recommended only as an interim therapy before curative treatment, such as GT or HSCT, even if such therapy is available to improve patient’s condition and prevent ongoing neuro- and hepatotoxicity (9). Recommended ERT duration ranges from few months to around 2 years, as ERT is associated with several adverse effects, including development of malignancies (often EBV-related lymphomas, mostly after 8-10 years of treatment), and ultimate failure due induction of enzyme neutralizing antibodies in about 10% of patients (1). An increase in B cell numbers is evident within the first month of treatment, and in T lymphocytes after 2-4 months, as was seen in our patients (19).

Costs of the drug and lack of reimbursement due to lack of drug registration in Poland and the European Union are the major obstacles in conducting ERT. As a consequence shortages in availability of ERT have been recorded in the past resulting in limited number of patients treated for short time.

## Conclusions

Despite low numbers of ADA-SCID patients diagnosed in Poland, an advancement in early diagnostics was observed due to an increase in physicians’ experience and improved availability of molecular diagnostics. Diagnosis in younger patients gives a chance for more effective treatment and prevention of irreversible damage.

ERT was effective in the analyzed cases, but high costs greatly limit access to the drug. Patients have restricted access to novel methods such as gene therapy, due to high cost and limitation to only few treatment centers.

Implementation of NBS for SCID in Poland could enable recognition of SCID, including ADA-SCID.

## Data availability statement

The original contributions presented in the study are included in the article/supplementary materials. Further inquiries can be directed to the corresponding author.

## Ethics statement

The studies involving human participants were reviewed and approved by Bioethics Committee at the Children’s Memorial Health Institute. Written informed consent to participate in this study was provided by the participants’ legal guardian/next of kin.

## Author contributions

ND-L, MP designed the concept of the manuscript. ND-L, BP, NB, MU, MP wrote the manuscript with contribution from all co-authors. BP did the immunological studies. ES did the adenosine deaminase activity. ND-L, KB-S, NB, EB, BW-K, KK, SK, AD, JG, MU, MP contributed to clinical data collection and critical review. All authors contributed to the article and approved the submitted version of the manuscript.
